# Seronegative Longitudinally Extensive Transverse Myelitis in a Filipino Adult: Diagnostic Challenges and Characteristic Longitudinal MRI Findings

**DOI:** 10.7759/cureus.112638

**Published:** 2026-07-14

**Authors:** Mary Grace S Aguda-Barillo, Jon Stewart H Dy, Jasmyn A De Leon

**Affiliations:** 1 Neurology, Chinese General Hospital and Medical Center, Manila, PHL; 2 Neurosciences, Mapua University School of Medicine, Makati City, PHL; 3 Neurosciences, St. Luke's Medical Center College of Medicine - William H. Quasha Memorial, Quezon City, PHL; 4 Neurology, Tanza Specialists Medical Center, Cavite, PHL; 5 Neurology, Cardinal Santos Medical Center, San Juan City, PHL

**Keywords:** autoimmune demyelinating disease, diagnostic and therapeutic challenge, longitudinally extensive transverse myelitis, magnetic resonance imaging of the spine, seronegative inflammatory myelopathy

## Abstract

Longitudinally extensive transverse myelitis (LETM) is a radiologic pattern of longitudinal spinal cord involvement most commonly associated with antibody-mediated autoimmune diseases such as neuromyelitis optica spectrum disorder and myelin oligodendrocyte glycoprotein antibody-associated disease. Seronegative inflammatory cases remain diagnostically challenging because they require careful exclusion of infectious, autoimmune, vascular, neoplastic, metabolic, and structural etiologies. A 45-year-old Filipino female presented with a three-day history of severe upper back pain, followed by rapidly progressive bilateral upper and lower extremity weakness, sensory disturbances, and urinary retention. Neurologic examination revealed asymmetric quadriparesis, a sensory level at the fourth thoracic dermatome, preserved vibration and proprioception, and mixed hyperreflexia and hyporeflexia with bilateral Babinski signs. Spinal magnetic resonance imaging (MRI) demonstrated a poorly defined, non-expansile intramedullary T2-hyperintense lesion extending contiguously from C3 to T2 without discrete enhancement. Diffusion-weighted imaging demonstrated no restricted diffusion. Cerebrospinal fluid analysis showed mild lymphocytic pleocytosis with normal IgG index and absence of oligoclonal bands. Extensive infectious, inflammatory, autoimmune, and metabolic investigations were unrevealing, including negative serum aquaporin-4 and myelin oligodendrocyte glycoprotein antibodies. Follow-up spinal MRI performed 3.5 months later demonstrated persistent longitudinal non-enhancing intramedullary T2 hyperintensity with evolution toward chronic myelomalacic change, providing important longitudinal imaging evidence supporting an inflammatory antibody-negative myelopathy over competing diagnostic considerations. The patient was treated with high-dose intravenous methylprednisolone, followed by an oral prednisone taper and subsequent initiation of azathioprine because of persistent severe deficits. Despite immunotherapy and rehabilitation, she remained wheelchair-dependent with persistent dysesthesias and neurogenic bladder at follow-up. Following comprehensive multidisciplinary evaluation and longitudinal follow-up, an inflammatory antibody-negative LETM was considered the most likely diagnosis after exclusion of competing etiologies. This case highlights the diagnostic complexity, therapeutic challenges, and potentially severe neurologic outcomes associated with seronegative LETM. It also underscores the importance of systematic exclusion of alternative etiologies, longitudinal follow-up, repeat serologic evaluation, and individualized consideration of escalation immunotherapy in patients with incomplete response to corticosteroid treatment.

## Introduction

Transverse myelitis is an inflammatory disorder of the spinal cord characterized by acute or subacute motor, sensory, and autonomic dysfunction [1]. When spinal cord involvement extends contiguously across three or more vertebral segments, the condition is classified as longitudinally extensive transverse myelitis (LETM), a radiologic pattern most commonly associated with antibody-mediated autoimmune diseases such as neuromyelitis optica spectrum disorder (NMOSD) and myelin oligodendrocyte glycoprotein-associated disease (MOGAD), although it may also occur in vascular, infectious, neoplastic, metabolic, and other inflammatory conditions [2-4]. Recognition of LETM is clinically important, as it often implies a more severe disease course and necessitates prompt evaluation to identify the underlying etiology and initiate appropriate therapy [4].

Despite advances in serologic testing, a subset of patients with inflammatory LETM remains negative for aquaporin-4 and myelin oligodendrocyte glycoprotein (MOG) antibodies after extensive evaluation [2,3]. These seronegative cases present a significant diagnostic challenge, as they require careful exclusion of infectious, systemic autoimmune, paraneoplastic, metabolic, structural, and vascular causes of longitudinally extensive myelopathy [4]. Moreover, the absence of disease-specific antibodies complicates prognostication and long-term management decisions, including relapse risk assessment and the need for maintenance immunosuppression [5].

Here, we report a case of seronegative LETM in a middle-aged Filipino female who presented with rapidly progressive quadriparesis and autonomic dysfunction. Following comprehensive clinical, laboratory, radiologic, and longitudinal evaluation, an inflammatory antibody-negative LETM was considered the most likely diagnosis after exclusion of competing etiologies. This case highlights the diagnostic complexity of antibody-negative inflammatory myelopathies and underscores the importance of a systematic multidisciplinary approach to evaluation and follow-up, particularly in patients with severe neurological deficits and incomplete recovery despite early immunotherapy.

## Case presentation

A 45-year-old Filipino female presented with a three-day history of sudden-onset, severe, and persistent upper back pain, followed by rapidly progressive neurological symptoms. She initially developed bilateral upper-extremity numbness and weakness, which subsequently progressed to involve both lower extremities and was accompanied by urinary retention. There was no preceding febrile illness, recent infection, vaccination, or toxin exposure. The patient specifically denied any history of recent falls, cervical manipulation, strenuous physical exertion, or other traumatic events preceding symptom onset. Her past medical history was significant for migraine without aura, well-controlled type 2 diabetes mellitus, and a nonfunctional pituitary macroadenoma. There was no prior history of demyelinating disease, systemic autoimmune disorder, malignancy, or spinal cord pathology.

On neurologic examination, the patient was awake, alert, and oriented to person, place, and time, with a Glasgow Coma Scale score of 15. There were no cranial nerve deficits. Motor examination revealed asymmetric quadriparesis, with Medical Research Council (MRC) grade 0/5 strength in the left upper and lower extremities, 3/5 strength in the proximal right upper and lower extremities, and 0/5 strength in the distal right upper and lower extremities [6]. Sensory examination demonstrated a last normal pinprick sensation at the fourth thoracic dermatome, with approximately 50% reduction in pinprick sensation from the fifth thoracic dermatome caudally. Vibration and joint position sense were preserved in all extremities. Deep tendon reflexes were asymmetric, with hyperreflexia noted in the right biceps, brachioradialis, triceps, and patellar reflexes, as well as the left patellar reflex, while the left biceps, brachioradialis, and triceps reflexes were hyporeflexic. Plantar responses were extensor bilaterally. Autonomic involvement was evident, with urinary retention requiring bladder catheterization.

**Figure 1:**
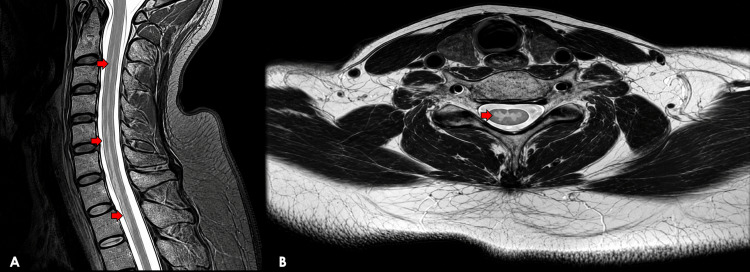
Cervicothoracic spine MRI demonstrating a longitudinally extensive intramedullary myelopathy. (A) Sagittal T2-weighted image demonstrates a poorly marginated, non-expansile T2-hyperintense intramedullary lesion extending contiguously from C3 to T2 (red arrows). (B) Axial T2-weighted image demonstrates predominant central intramedullary involvement without significant spinal cord enlargement, focal mass effect, or compressive pathology (red arrow). Diffusion-weighted imaging demonstrated no restricted diffusion, and no discrete intramedullary enhancement was identified on post-contrast imaging.

Magnetic resonance imaging (MRI) of the spine (Figure 1), performed on day 3 after symptom onset, demonstrated a longitudinally extensive, poorly marginated, non-expansile intramedullary T2-hyperintense lesion extending contiguously from the third cervical to the second thoracic spinal cord levels. The lesion predominantly involved the central spinal cord without evidence of focal mass effect or significant cord enlargement. Diffusion-weighted imaging demonstrated no restricted diffusion. No discrete intramedullary enhancement was identified on post-contrast sequences. These findings were most consistent with an inflammatory, longitudinally extensive myelopathy.

**Figure 2:**
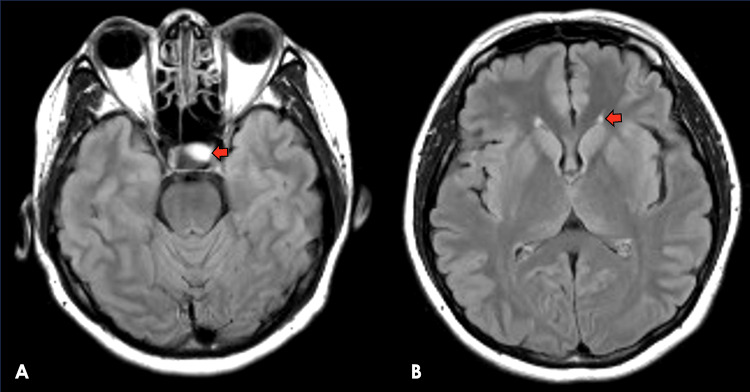
Axial fluid-attenuated inversion recovery (FLAIR) MRI of the brain demonstrates a known left-sided pituitary macroadenoma (A, red arrow) and a few chronic small-vessel ischemic white matter changes (B, red arrow). No imaging features suggestive of central nervous system demyelination are identified.

MRI of the brain with contrast (Figure 2) demonstrated a known left-sided pituitary macroadenoma and mild chronic small-vessel ischemic changes without evidence of acute infarction, enhancing lesions, or demyelinating brain lesions suggestive of multiple sclerosis (MS) or other central inflammatory disorders.

Cerebrospinal fluid (CSF) analysis (Table 1) revealed mild pleocytosis (6.7 cells/µL) with lymphocytic predominance. CSF protein and glucose levels were within normal limits. The CSF IgG index was normal, and oligoclonal bands were absent. CSF studies were negative for infectious etiologies.

**Table 1:** Cerebrospinal fluid analysis. cm H2O = centimeter water; mm3 = cubic millimeters; mg/dL = milligrams per deciliter; CSF = cerebrospinal fluid; VDRL = Venereal Disease Research Laboratory; IgG = immunoglobulin G.

Test	Results	Reference value
Opening pressure	Not measured due to slow flow	8-18 cm H2O
Closing pressure	Not measured due to slow flow	8-18 cm H2O
Gross description	Clear and free-flowing	Clear and free-flowing
Cells	6.7	0-5 cells/mm3
Neutrophils	30%	Not applicable
Lymphocytes	70%	Not applicable
CSF-serum glucose	0.7	0.5-0.6
Protein	28.2	8-32 mg/dL
Gram stain	Negative	Negative
Culture and sensitivity	Negative for 3 days	Negative
Japanese B encephalitis virus, dengue virus, SARS-CoV-2	Negative	Negative
Acid-fast bacilli	Negative	Negative
Mycobacterium tuberculosis polymerase chain reaction	Negative	Negative
Tuberculosis culture and sensitivity	Negative for 42 days	Negative
Potassium hydroxide	Negative	Negative
Cryptococcal antigen latex agglutination system	Negative	Negative
India Ink	Negative	Negative
VDRL	Negative	Negative
Film array (Escherichia coli, Haemophilus influenzae, Listeria monocytogenes, Neisseria meningitidis, Streptococcus pneumoniae, Streptococcus agalactiae, Cryptococcus neoformans, Cytomegalovirus, Enterovirus, Herpes simplex virus 1, Herpes simplex virus 2, Human herpesvirus 6, Human parechovirus, Varicella-zoster virus)	Negative	Negative
Oligoclonal bands	Negative	Negative
IgG	1.5	<6 mg/dL
IgG index	0.27	<0.7
Cell block	Negative	Negative
Cytology	Negative	Negative

Extensive serum evaluation (Table 2) for inflammatory, autoimmune, and infectious causes of myelopathy was unremarkable. Serum aquaporin-4 immunoglobulin G and MOG antibodies tested via cell-based assay were negative. Autoimmune screening, including antinuclear antibody, perinuclear antineutrophil cytoplasmic antibody, cytoplasmic antineutrophil cytoplasmic antibody, and antiphospholipid antibody panel, was likewise negative. Routine laboratory investigations did not reveal metabolic or systemic abnormalities to suggest an alternative etiology.

**Table 2:** Laboratory tests done during admission. g/dL = grams per deciliter; mm3 = cubic millimeters; fL = femtoliter; mg/dL = milligrams per deciliter; mmol/L = millimoles per liter; U/L = units per liter; CRP = C-reactive protein; C3 = complement component 3; C4 = complement component 4; anti-HBsAg = hepatitis B surface antigen antibody; HBsAg = hepatitis B surface antigen; HIV = human immunodeficiency virus; ANA = antinuclear antibody; p-ANCA = perinuclear antineutrophil cytoplasmic antibody; c-ANCA = cytoplasmic antineutrophil cytoplasmic antibody; IgM = Immunoglobulin M; IgG = Immunoglobulin G.

Test	Results	Reference value
Hemoglobin	12.2	11.6-15.5 g/dL
Hematocrit	37.0	36.0-47.0%
White blood cell count	^9,600^	4,800-10,800 cells/mm^3^
Neutrophils	89	40-74%
Lymphocytes	6	19-48%
Monocytes	5	3-9%
Platelet count	207,000	150,000-400,000/mm^3^
Mean corpuscular volume	84.8	82-98 fL
Mean corpuscular hematocrit	27.6	28-33 fL
Mean corpuscular hemoglobin concentration	32	32-38%
Creatinine	0.74	0.55-1.2 mg/dL
Sodium	139	136-145 mmol/L
Potassium	3.8	3.5-5.1 mmol/L
Alanine aminotransferase	58	10-49 U/L
Aspartate aminotransferase	28	0-34 U/L
Procalcitonin	0.01	<0.05 ng/mL
CRP	0.11	<1 mg/dL
C3	1,056.65	800-1850 mg/L
C4	219.74	100-400 mg/L
Anti-HBsAg	Nonreactive	Nonreactive
HBsAg	Nonreactive	Nonreactive
HIV antigen and antibody	Nonreactive	Nonreactive
Total protein	53	60-83 g/L
Albumin	35	35-50 g/L
Globulin	18	20-35 g/L
ANA	Negative	Negative
Anti-aquaporin 4 antibody	Negative	Negative
Anti-MOG antibody	Negative	Negative
p-ANCA	Negative	Negative
c-ANCA	Negative	Negative
Anticardiolipin IgM	Negative	Negative
Anticardiolipin IgG	Negative	Negative

Following multidisciplinary evaluation integrating the clinical presentation, neuroimaging, CSF analysis, and extensive laboratory investigations, inflammatory LETM was considered the most likely diagnosis. Important alternative diagnoses considered included NMOSD [7], MOGAD [8], MS [9], spinal cord infarction, infectious myelitis, systemic autoimmune disease, and neoplastic, metabolic, vascular, and compressive myelopathies. Spinal cord infarction remained an important differential diagnosis because of the abrupt onset of neurological deficits. However, the absence of restricted diffusion on spinal diffusion-weighted imaging, lack of a characteristic anterior spinal artery territorial imaging pattern, absence of an identifiable vascular precipitating event (including hypotension, vascular intervention, or aortic pathology), and persistent non-enhancing longitudinal T2 hyperintensity with radiologic features favoring chronic myelomalacic change on follow-up MRI performed 3.5 months later favored an inflammatory longitudinally extensive myelopathy, making spinal cord infarction considerably less likely. No evidence of compressive spinal cord pathology, infiltrative disease, or systemic malignancy was identified.

Overall, the clinical presentation, longitudinal neuroimaging findings, CSF profile, and exclusion of alternative etiologies were considered most consistent with seronegative LETM [10].

The patient was treated with high-dose intravenous methylprednisolone at a cumulative dose of 5 g administered over five consecutive days. This was followed by oral prednisone 60 mg daily for four weeks, with a planned gradual taper. Supportive management included bladder catheterization for urinary retention and initiation of inpatient rehabilitation, followed by ongoing outpatient physiotherapy.

Following completion of corticosteroid therapy, the patient demonstrated only partial neurological improvement. Severe quadriparesis persisted with limited recovery of motor strength. She remained wheelchair-dependent and continued to experience dysesthesias involving all four extremities. Autonomic dysfunction also persisted, with urinary retention requiring long-term bladder catheterization.

**Figure 3:**
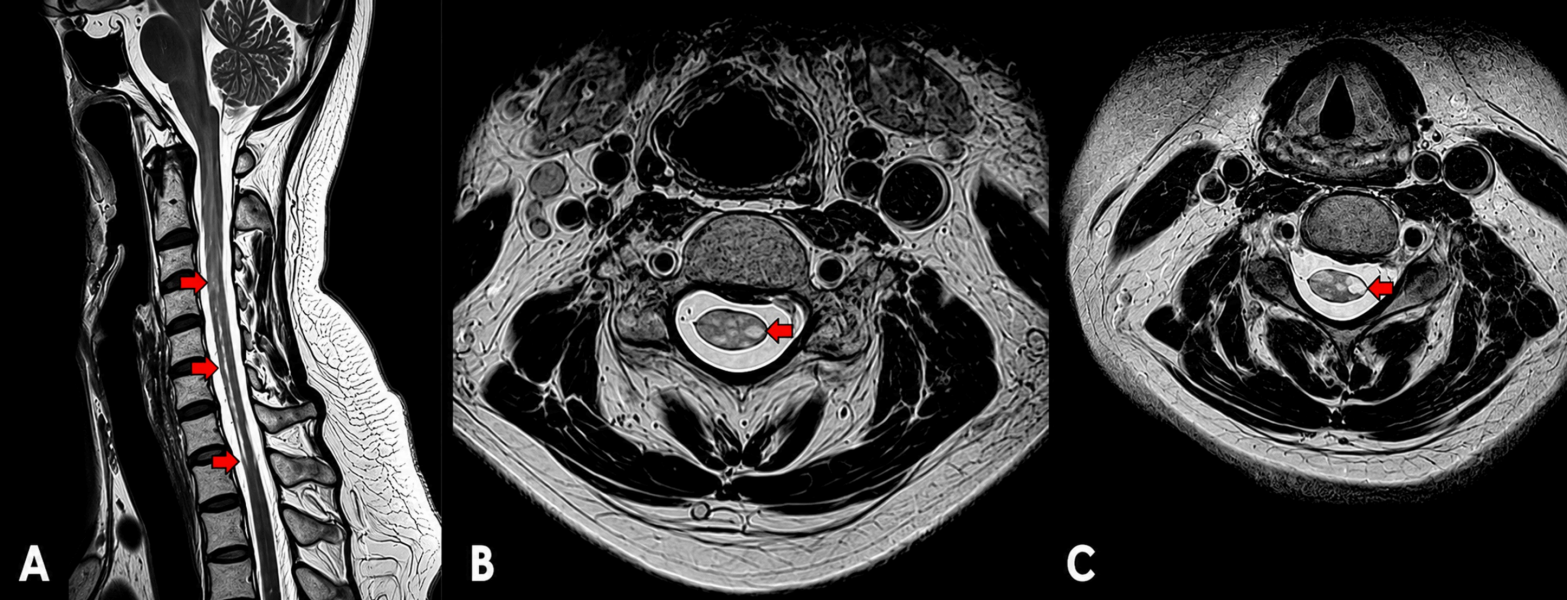
Follow-up cervical spine MRI obtained 3.5 months after symptom onset. (A) Sagittal T2-weighted image demonstrating persistent longitudinal non-enhancing intramedullary T2 hyperintensity extending from approximately C2 to T1 (red arrows) without significant interval change compared with the baseline MRI. (B-C) Representative axial T2-weighted images demonstrating persistent central and left-sided intramedullary T2 hyperintensity (red arrows). The lesions appear more well-defined on follow-up imaging, with radiologic features favoring chronic myelomalacic change.

The patient subsequently underwent structured inpatient and outpatient rehabilitation, followed by physiotherapy three times weekly. At the most recent follow-up, 3.5 months after symptom onset, repeat cervical spine MRI (Figure 3) demonstrated persistent non-enhancing longitudinal intramedullary T2 hyperintensity extending from C2 to T1 without significant interval change compared with the initial examination. The lesion appeared more well-defined on follow-up imaging, with radiologic features favoring chronic myelomalacic change. Repeat brain and orbital MRI with contrast remained unchanged, without new intracranial, optic nerve, or enhancing lesions. No clinical relapse occurred; however, marked functional impairment remained. The patient continued tapering oral prednisone. Because of the severity of residual neurological deficits and incomplete response to corticosteroids, escalation of immunotherapy was considered. Plasma exchange was contemplated for steroid-refractory disease but could not be performed because of logistical and resource limitations during the acute hospitalization. The patient was subsequently initiated on azathioprine 50 mg twice daily as a steroid-sparing immunosuppressive agent for presumed antibody-negative autoimmune inflammatory myelopathy. Repeat serologic testing for NMOSD and MOGAD was also planned, recognizing that false-negative antibody results may occur early in the disease course or following immunotherapy exposure. The chronology of the patient’s presentation, diagnostic workup, treatment, and follow-up is summarized in Table 3.

**Table 3:** Clinical timeline of presentation, diagnostic evaluation, treatment, and follow-up. Day 0 = day of hospitalization; IgG = immunoglobulin G.

Timeline	Clinical events
Day -3	Onset of severe upper back pain
Day -2 to -1	Progressive bilateral upper extremity numbness and weakness, followed by lower extremity involvement
Day 0	Development of urinary retention and hospital admission
Day 0	Neurologic examination demonstrated asymmetric quadriparesis, fourth thoracic dermatomal sensory level, mixed hyperreflexia/hyporeflexia, and bilateral Babinski signs
Day 0-1	MRI of the cervicothoracic spine demonstrated a longitudinally extensive intramedullary T2-hyperintense lesion extending from the third cervical to the second thoracic spinal levels
Day 1	Cerebrospinal fluid analysis showed mild lymphocytic pleocytosis with a normal IgG index and absent oligoclonal bands
Hospitalization period	Extensive infectious, autoimmune, inflammatory, and metabolic investigations were performed; serum aquaporin 4-IgG and MOG antibodies were negative
Days 1-5	Treated with high-dose intravenous methylprednisolone (total cumulative dose: 5 grams over 5 days)
Post-steroid treatment	Partial neurologic improvement but persistent severe quadriparesis and autonomic dysfunction
Discharge period	Initiation of oral prednisone taper and structured rehabilitation program
3.5 months follow-up	Persistent wheelchair dependence, dysesthesias, and neurogenic bladder; repeat spinal MRI showed no interval improvement
Follow-up period	Azathioprine initiated for steroid-sparing immunosuppression and possible relapse prevention; repeat testing for serum aquaporin 4-IgG and MOG antibodies planned

## Discussion

LETM is a radiologic pattern of spinal cord involvement most frequently associated with antibody-mediated autoimmune disorders, particularly NMOSD and MOGAD [2-5,11]. Recognition of LETM is clinically important because it often reflects severe spinal cord involvement requiring prompt diagnostic evaluation and, when an inflammatory etiology is suspected, timely immunotherapy. However, LETM is not disease-specific and may also occur in vascular, infectious, neoplastic, metabolic, and other inflammatory disorders [4,11]. Consequently, antibody-negative LETM remains a diagnosis of exclusion requiring systematic evaluation of competing etiologies [4,11].

In the present case, extensive investigations failed to identify an infectious, systemic autoimmune, neoplastic, metabolic, vascular, or structural cause of the patient’s myelopathy. Spinal cord infarction represented the principal competing diagnosis, given the abrupt onset of severe neurological deficits. We agree that spinal cord infarction can closely mimic inflammatory LETM clinically and radiologically, particularly during the acute phase [4]. Although diffusion-weighted imaging may improve diagnostic confidence, its sensitivity remains imperfect because of technical limitations and the relatively small size of the spinal cord [4]. Nevertheless, several features favored an inflammatory etiology in our patient. The lesion morphology was not typical of an anterior spinal artery territorial infarction. Diffusion-weighted imaging demonstrated no restricted diffusion, no vascular precipitating event (including hypotension, vascular intervention, or aortic pathology), and no major vascular risk factors were identified. Follow-up MRI performed 3.5 months later demonstrated persistent non-enhancing longitudinal T2 hyperintensity without significant interval evolution, with radiologic features favoring chronic myelomalacic change. Taken together with the mild CSF pleocytosis and the overall clinicoradiologic findings, inflammatory longitudinally extensive myelopathy remained the favored diagnosis, with the longitudinal imaging findings making spinal cord infarction less likely. Repeat brain and orbital MRI with contrast likewise remained unchanged and demonstrated no interval development of intracranial or optic nerve lesions suggestive of NMOSD, MOGAD, or MS. This case illustrates the importance of integrating clinical presentation, serial neuroimaging, laboratory investigations, and longitudinal follow-up when distinguishing inflammatory from ischemic myelopathies.

The absence of serum aquaporin-4 and MOG antibodies, lack of characteristic brain MRI findings suggestive of MS, and CSF profile not strongly supportive of a classical demyelinating disorder favored a diagnosis of seronegative LETM [2,3,9]. Nevertheless, antibody-negative inflammatory myelopathies remain diagnostically challenging because serologic assays may yield false-negative results early in the disease course or following immunosuppressive therapy, particularly after corticosteroid exposure [12]. Accordingly, repeat antibody testing and continued longitudinal clinical surveillance remain important in patients with severe or atypical presentations [12].

Additional investigations, including coagulation studies, serum anti-HTLV-1 serology, and CSF glial fibrillary acidic protein (GFAP) antibody testing, may further aid the evaluation of selected patients with longitudinally extensive myelopathy but were not available in this case. HTLV-1-associated myelopathy may rarely present with acute or subacute longitudinally extensive spinal cord lesions and should be considered in appropriate epidemiologic settings [13]. Although anti-HTLV-1 antibody testing was unavailable, the patient’s abrupt presentation, absence of systemic manifestations, and overall clinicoradiologic findings made this diagnosis less likely.

Other inflammatory etiologies, including neurosarcoidosis, GFAP astrocytopathy, and paraneoplastic myelopathies, may also present with longitudinally extensive spinal cord lesions and should be considered in antibody-negative cases [4,11]. In our patient, the absence of systemic manifestations, lack of characteristic imaging findings, negative CSF cytology, and unrevealing autoimmune and infectious investigations reduced the likelihood of these alternative diagnoses. Nonetheless, the evolving understanding of autoimmune myelopathies highlights the importance of continued reassessment in patients with persistent or progressive deficits.

The patient’s incomplete neurological recovery despite early administration of high-dose corticosteroids underscores the potential severity of antibody-negative inflammatory myelopathy. Poor prognostic factors include longitudinally extensive lesions, profound motor deficits at nadir, and early autonomic dysfunction, all of which were present in this patient [14]. These features likely contributed to the patient’s persistent disability and continued dependence on assistive care.

Management of seronegative LETM remains challenging because evidence-based treatment strategies are limited and are largely extrapolated from NMOSD and MOGAD treatment paradigms [2,3,11]. High-dose intravenous corticosteroids remain first-line therapy for acute inflammatory myelitis, whereas plasma exchange or intravenous immunoglobulin is recommended in patients with severe deficits or inadequate response to corticosteroids [15]. In this patient, plasma exchange was considered but was not feasible because of resource limitations during the acute hospitalization. Given the severity of persistent neurological deficits and the possibility of an underlying antibody-negative autoimmune inflammatory myelopathy, azathioprine was initiated as a steroid-sparing immunosuppressive agent while repeat serologic evaluation remained planned. Observational studies suggest that some patients with double-seronegative LETM experience severe disability and may subsequently relapse, supporting consideration of maintenance immunosuppression in selected patients [16].

This case highlights the diagnostic complexity of antibody-negative longitudinally extensive myelopathy and emphasizes that seronegative LETM remains a diagnosis of exclusion requiring careful differentiation from inflammatory, vascular, infectious, neoplastic, metabolic, traumatic, and compressive myelopathies. Comprehensive multidisciplinary evaluation, serial clinical, neuroimaging, and serologic follow-up are essential for establishing the most likely diagnosis and guiding individualized management. Further studies are needed to better define the pathophysiology, optimal treatment strategies, and long-term outcomes of seronegative inflammatory myelopathies.

## Conclusions

Seronegative LETM remains a diagnostically challenging clinical entity that requires systematic exclusion of inflammatory, infectious, vascular, traumatic, metabolic, neoplastic, and structural causes of longitudinally extensive myelopathy, together with careful longitudinal assessment. This case illustrates that severe inflammatory myelopathy may occur despite negative aquaporin-4 and MOG antibody testing and may be associated with poor neurological recovery despite timely corticosteroid therapy. Identification of adverse prognostic features, including longitudinally extensive spinal cord involvement, profound motor deficits, and early autonomic dysfunction, is important for guiding management and patient counseling. Comprehensive multidisciplinary evaluation, serial neuroimaging, repeat serologic assessment, and ongoing clinical follow-up remain essential for establishing the most likely diagnosis and guiding individualized management in patients with antibody-negative longitudinally extensive myelopathy.
